# 57. Evaluation of the 2019 European Heart Rhythm Association International Consensus Document in Patients with Cardiovascular Implantable Electronic Devices Who Develop *Staphylococcus aureus* Bacteremia

**DOI:** 10.1093/ofid/ofab466.057

**Published:** 2021-12-04

**Authors:** Supavit Chesdachai, Larry M Baddour, Muhammad R Sohail, Raj Palraj, Malini Madhavan, Hussam Tabaja, Madiha Fida, Daniel DeSimone

**Affiliations:** 1 Mayo Clinic, Rochester, MN; 2 Mayo Clinic College of Medicine, Rochester, MN; 3 Baylor College of Medicine, Houston, Texas

## Abstract

**Background:**

Cardiovascular implantable electronic device (CIED) implantation has markedly increased over the past two decades. *Staphylococcus aureus* bacteremia (SAB) occurs in patients with CIED and determination of device infection among patients without clinical findings of pocket site infection is often difficult. Our study examines the characteristics, management, and outcomes of SAB in patients living with CIED using 2019 international criteria to define CIED infection.

**Methods:**

We conducted a retrospective study of patients with CIED who were hospitalized at Mayo Clinic, Rochester, with SAB from January 1, 2012 to December 31, 2019. Patients who met CIED infection criteria following SAB based on the 2019 European Heart Rhythm Association International Concensus Document were identified. A time-to-event analysis was used to determine the impact, if any, of complete device extraction on outcomes.

**Results:**

Overall, 110 patients with CIED developed SAB and 92 (83.6%) of them underwent transesophageal echocardiogram (TEE). Eighty-eight (80%) had CIED infection with 57 (51.8%) and 31 (28.2%) patients meeeting criteria for definite and possible CIED infections, respectively. Forty-three (75.4%) patients with definite CIED infection underwent complete device extraction. For possible and rejected CIED infection, the rates of complete device extraction were 35.5% and 27.3%, respectively (p< .001 for each). The primary endpoint of a composite of one-year mortality and SAB relapse had a rate that was significantly lower in patients with CIED infection who underwent complete device extraction as compared to that of patients who did not undergo device extraction (25.9% vs. 76.5%, p< .001). No significant difference in outcomes was seen in the rejected CIED infection group (33.3% vs. 62.5%, p =.27).

**Conclusion:**

The rate of CIED infections following SAB was higher than that reported previously. Increased use of TEE and a novel case definition with broader diagnostic criteria were likely operative, in part, in accounting for the the higher rate of CIED infections complicating SAB. Complete device removal is critical in patients with either definite or possible CIED infection as defined by the 2019 consensus document to improve one-year mortality and SAB relapse rates.

**Disclosures:**

**Larry M. Baddour, MD**, Boston Scientific (Individual(s) Involved: Self): Consultant; Botanix Pharmaceuticals (Individual(s) Involved: Self): Consultant; Roivant Sciences (Individual(s) Involved: Self): Consultant **Muhammad R. Sohail, MD**, **Medtronic** (Consultant)**Philips** (Consultant)

